# Reinvestigation of the crystal structure of Ca_2_Ce_8_(SiO_4_)_6_O_2_ apatite by Rietveld refinement

**DOI:** 10.1107/S2056989018008435

**Published:** 2018-06-12

**Authors:** Nicolas Massoni, Ronan Hegron, Lionel Campayo

**Affiliations:** a CEA, DEN, DE2D, Marcoule, BP17171, F-30207 Bagnols sur Ceze, France

**Keywords:** crystal structure, powder diffraction, cerium calcium silicate oxide apatite, redetermination

## Abstract

Ca_2_ *Ln*_8_(SiO_4_)_6_O_2_ apatites with *Ln* = La, Ce, Pr, Nd, Sm, Eu, Gd and Tb crystallize in space group *P*6_3_/*m*. The crystal structure of apatite-type Ca_2_Ce_8_(SiO_4_)_6_O_2_ [dicalcium octa­cerium hexa­kis­(silicate) dioxide], which has been synthesized by calcination, was refined from powder X-ray diffraction data using the Rietveld method. A database survey shows that contrary to the previously published Ca_2_Ce_8_(SiO_4_)_6_O_2_ structure [Skakle *et al.* (2000 ▸). *Powder Diffr.* **15**, 234–238], the cell volume of the structure reported here is consistent with those of other *Ln* apatites.

## Abstract 2

The lattice parameters of apatite-type Ca_2_Ca_8_(SiO_4_)_6_O_2_ determined from X-ray powder diffraction data are inconsistent with those found in the literature for the same type of material. Hence the structure was redetermined and compared with previously published data.

## Chemical context   

Contaminated metallic wastes produced by the nuclear industry need to be managed. This is often achieved by melting them with an oxide slag to make the waste packages more dense and to concentrate the residual plutonium or uranium oxide contamination of the metal. Laboratory work on actinides is facilitated by the use of surrogates that mimic their properties of inter­est. Cerium can thus be used to simulate the presence of plutonium (Ramsey *et al.*, 1995 ▸). The reactivity of cerium(IV) oxide added to melted stainless steel and an SiO_2_–CaO–Al_2_O_3_ glass slag under neutral conditions and at high temperature was studied. Powdered stainless steel from Alfa Aesar and an SiO_2_–CaO–Al_2_O_3_ lab-made glass frit were fused at 1723 K under argon for 6 h in a graphite crucible. Cerium(IV) oxide was introduced to simulate PuO_2_. The Ellingham diagram predicts that Ce^IV^ is reduced to Ce^III^ under these conditions, which is the predominant cerium form at 1723 K. The same behavior is expected for Pu (Ellingham, 1944 ▸). After melting, SEM observations of the sample showed that cerium was concentrated in the glass, as expected, but in two different forms. Cerium is present in the homogeneous part of the glass, at a typical content of 5 *wt*.% but is also found inside large crystals of hundreds of micrometers across. The X-ray diffractogram of bulk material shows an amorphous bump, attributed to the glass, and a typical pattern for an apatite structure in space group type *P*6_3_/*m*. The approximate composition of this phase was determined as Ca_2.4±0.3_Ce_7.6±0.3_(SiO_4_)_6_O_2_ and a cell volume of roughly 560 Å^3^. However, the diffraction data is inconsistent with the PDF card for Ca_2_Ce_8_(SiO_4_)_6_O_2_ (#00-055-0835; ICDD, 2015 ▸) with a cell volume of 530.96 Å^3^ (Skakle *et al.*, 2000 ▸). The difference between the two volumes, 5.2%, cannot be explained by the difference in composition between the two phases. For instance, the cell volumes of the apatites Ca_2_Nd_8_(SiO_4_)_6_O_2_ (#00-028-0228) and Ca_2.8_Nd_7.8_(SiO_4_)_6_O_2_ (#04-007-5969) are 552.20 and 551.76 Å^3^, respectively, a difference of just 0.08%. Moreover, the difference between the cell volumes of Ca_2_La_8_(SiO_4_)_6_O_2_ (#00-029-0337) and Ca_4_La_6_(SiO_4_)_6_O_2_ (#04-007-9090) is 0.3%. In other words, the 5.2% difference between the cell volume of Ca_2_Ce_8_(SiO_4_)_6_O_2_ (Skakle *et al.*, 2000 ▸) and Ca_2.4±0.3_Ce_7.6±0.3_(SiO_4_)_6_O_2_ (this work) cannot be explained by their differing calcium contents. These are the reasons why the structure of Ca_2_Ce_8_(SiO_4_)_6_O_2_ was reinvestigated in the present work.

## Structural commentary   

Apatites are mineral phases whose general formula is *A*_10_(*X*O_4_)_6_ *Z*_2_, where *A* = Ca, Sr, Ba, or many rare earth elements, *X* = B, Si, P, V, As and *Z* = OH, Cl, F, O, *etc*. (Byrappa &Yoshimura, 2001 ▸). Generally, apatites crystallize in the hexa­gonal crystal system in space group *P*6_3_/*m*. There are two types of *A* cations in these structures: Type I (Wyckoff position 4*f*) *A* cations are aligned along the threefold rotation axes. Theses cations are separated on each of these axes by one-half the value of the *c* parameter. Type I cations are sometimes called columnar cations. They are ninefold coordinated by oxygen atoms and these columns of *A*O_9_ polyhedra are linked together by *X*O_4_ tetra­hedra, with three of the oxygen atoms belonging to one column and the fourth to an adjacent column (Elliott, 1994 ▸). This results in a skeleton of *X*O_4_ tetra­hedra (point group symmetry *m*..) alongside the columnar *A* cations. This skeleton defines channels that are collinear to the *c* axis and which correspond to the sixfold screw axes. The *Z* anions and the remaining *A* cations, also called type II cations and located on mirror planes (6*h*), are located inside these channels with the *Z* anions positioned in ellipsoidal cavities along the *c* axis. Type II cations are sevenfold coordinated, and these sites are smaller than those centered on type I cations. For the present structure, type I cations are statistically occupied by Ca and Ce whereas type II cations are solely occupied by Ce.

**Figure 1:**
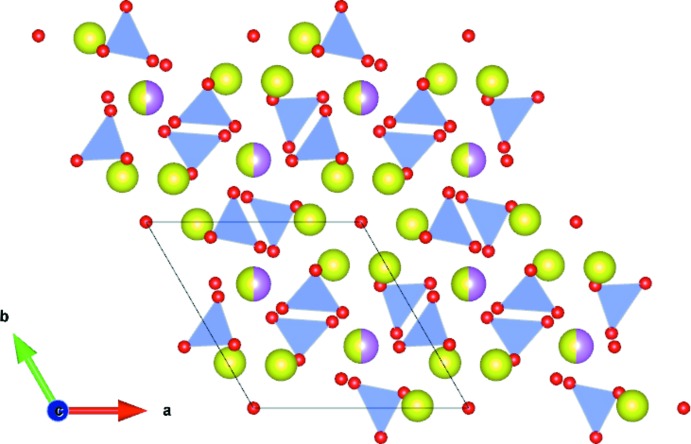
Polyhedral representation of the Ca_2_Ce_8_(SiO_4_)_6_O_2_ structure. SiO_4_ tetra­hedra are in blue, the mixed Ca/Ce sites are shown as pink/yellow and the Ce sites as yellow spheres, respectively.

The refined title structure is displayed in Fig. 1 ▸. In the case of the synthesized Ca_2_Ce_8_(SiO_4_)_6_O_2_ apatite, the possible substitution of trivalent Ce by tetra­valent Ce would require one positive charge, which should be balanced by replacing calcium by a monovalent cation. No such element was detected by energy-dispersive X-ray fluorescence (EDS). Such a substitution of Ce^3+^ by Ce^4+^ in a britholite was investigated by Terra (2005 ▸). However, the characterization of the substitution gave unclear results, revealing that the substitution was not successful. This indicates that the presence of tetra­valent Ce in the synthesized apatite is also unlikely. It should be noted that there is no obvious explanation for the differences between our structure model (in particular in terms of lattice parameters) and the structure model with the same composition reported by Skakle *et al.* (2000 ▸; #00-055-0835). These authors stated that the difference might result from a different synthesis route, *i.e.* hydro­thermal for their Ce-apatite versus solid-state routes for other *Ln*-apatites. However, in our opinion, differences in the synthesis route can result only in slight differences between the resulting structures for a given composition. On the other hand, differences occur mainly because of slight variations in the composition, especially for wet synthesis routes for which the presence of carbonate or hydrogenphosphate in the structure can be difficult to avoid. Another known source of variations in the lattice volume is the presence of radiation defects, but this does not seem relevant here. Table 1 ▸ reports some bond lengths compared with the already published Ce-apatite #00-055-0835 (Skakle *et al.*, 2000 ▸) and La-apatite #00-029-0337 (Smith & McCarthy, 1977 ▸). As already noticed by Skakle *et al.* (2000 ▸), the Si1—O1 bond length of their structure model was rather short (1.42 Å), with the corresponding SiO_4_ tetra­hedron highly distorted, as shown by the distorsion index calculated by *VESTA* (Momma & Izumi, 2011 ▸). The Si—O bond lengths of the redetermined structure are in good agreement with expected values and those of other *La*-apatites (Table 1 ▸). Likewise, the distorsion index of the SiO_4_ tetra­hedron is smaller with an order of magnitude for the redetermined structure and other La-apatites.

## Database survey   

**Figure 2:**
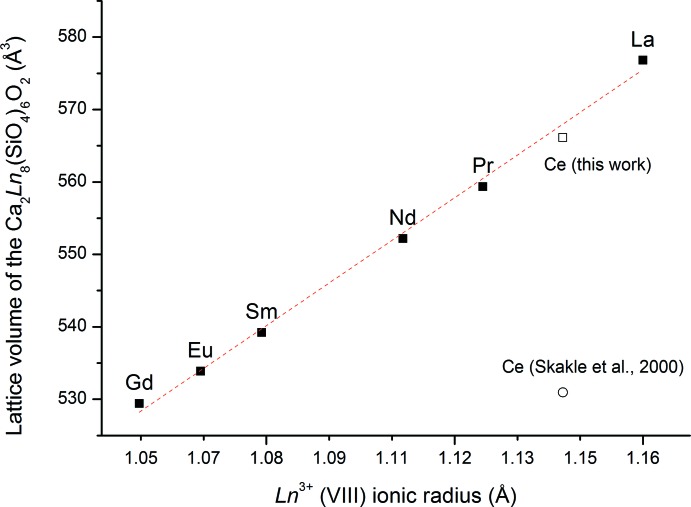
Plot of the volume of Ca_2_ *Ln* ^III^_8_(SiO_4_)_6_O_2_ *Ln*-apatites *versus* ionic radii of *Ln* ^3+^ ions in a VIII-coordinated environment.

The focus here is on silicate oxide apatites containing calcium and lanthanides. For most lanthanides, the structures published with the highest notation index are those of Ca_2_ *Ln*_8_(SiO_4_)_6_O_2_ apatites, indicating that they were refined in terms of lattice parameters and atomic positions. The cell parameters in apatite structure are highly dependent on the nature of the lanthanide. Structure data for La (#00-029-0337), Pr (#00-029-0362), Sm (#00-029-0365), Eu (#00-029-0320), Gd (#00-028-0212) apatites (Smith & McCarthy, 1977 ▸), as well as the Ce (#00-055-0835) (Skakle *et al.*, 2000 ▸) and the Nd (#00-028-0228) apatite (Fahey *et al.*, 1985 ▸) have been reported. Fig. 2 ▸ shows the ionic radius of *Ln* ^3+^ in a VIII-coordinated environment (Shannon, 1976 ▸) as a function of the volume of the *Ln*-apatite.

The linear correlation indicates that the ionic radius of the lanthanide controls the cell volume. For the Ce-apatite (#00-055-0835), the cell volume in the published structure is 530.96 Å^3^, which does not follow the general trend (Skakle *et al.*, 2000 ▸). However, the structure reported in the current article fits with the linear correlation between ionic radius and cell volume. Table 2 ▸ compiles the corresponding lattice parameters and cell volumes.

The database survey was extended to cerium-based *P*6_3_/*m* phases and identified a number of silicate apatites (also known as britholites) as possible matches. The Ce_9.33_(SiO_4_)_6_O_2_ phase, characterized by Rocchini *et al.* (2000 ▸), has a chemical composition similar to the structure reported here as it contains Ce^III^ without calcium (#00-054-0618, *a* = 9.598 Å, *c* = 7.106 Å, *V* = 566.91 Å^3^). Natural britolithe with composition Ca_4_(Ce,La,Nd,Ca,Th)_6_(SiO_4_)_6_O_2_ (#00-046-1294; *a* = 9.59 Å, *c* = 7.04 Å, *V* = 561.53 Å^3^) is a silicate apatite that contains a combination of rare earth elements with cerium (Orlandi *et al.*, 1989 ▸). The lattice parameters are very close to the values reported in this paper but this phase is too rich in calcium, *i.e*. 10.11 *wt*.% compared with 4.5 *wt*.% for the Ca_2_Ce_8_(SiO_4_)_6_O_2_ apatite. Oxyfluorinated silicate phases should also be considered. Within this compositional frame, the closest synthetic britolithe reported is cerium calcium strontium silicate fluoride oxide (#01-077-0619; *a* = 9.64 Å, *c* = 7.08 Å, *V* = 569.64 Å^3^) the formula of which is (Ce_0.4_Ca_0.35_Sr_0.25_)_4_(Ce_0.86_Ca_0.14_)_6_(SiO_4_)_6_(O_0.5_F_0.38_)_2_ (Genkina *et al.*, 1991 ▸). This apatite seems to be an isotype of the current redetermined structure because its calcium content is similar (5.26 *wt*.% *versus* 4.5 *wt*.%). However, this material differs from the one reported here because it contains Sr (5.13 *wt*.%) and fluorine (0.85 *wt*.%).

## Synthesis and crystallization   

The usual synthesis protocol for Ca_2_ *Ln*_8_(SiO_4_)_6_O_2_ apatites is a calcination of CaO, *Ln*_2_O_3_ and SiO_2_ in appropriate amounts under air above 1673 K (Nicoleau *et al.*, 2016 ▸). A trivalent *Ln* precursor is used, with the same oxidation state as found in the final apatite. However, cerium oxide is usually only available as CeO_2_, in which cerium is tetra­valent. The synthesis of Ce_2_O_3_ is known to be quite difficult and this phase is not fully stable under air (Bärnighausen & Schiller, 1985 ▸; Strydom & van Vuuren, 1987 ▸; Perrichon *et al.*, 1994 ▸; Hamm *et al.*, 2014 ▸). Hence, a particular synthesis protocol was adopted to successfully prepare the Ca_2_Ce_8_(SiO_4_)_6_O_2_ phase. Metallic silicon was added to a mixture of CeO_2_, SiO_2_ and CaO to ensure a double reaction during the thermal treatment: (i) *in situ* reduction of CeO_2_ to Ce_2_O_3_ with oxidation of Si to SiO_2_ and (ii) synthesis of the apatite by calcination of Ce_2_O_3_, SiO_2_ and CaO. Silicon was chosen because it reduces CeO_2_ to Ce_2_O_3_ and is inert to CaO, SiO_2_ and the alumina crucible used for the reaction (Ellingham, 1944 ▸). Moreover, the product of the reaction relates to the final composition of the apatite without by-products. The following amounts of precursors were used: 688.5 mg of CeO_2_, 28.1 mg of Si, 120.2 mg of SiO_2_ and 56.1 mg of CaO. The mixture was manually milled three times in an agate mortar and gently pressed in an alumina crucible. The sample was then heat treated under argon at 1873 K for 1 h with a heating ramp of 10 K min^−1^, then cooled at a controlled rate of 30 K min^−1^. A radially and axially shrunk pellet was obtained with a homogeneous brownish color, which was crushed in an agate mortar before X-ray diffraction measurements. The powdered sample was analyzed in a Panalytical XPert MPD Pro diffractometer in Bragg Brentano geometry for 5 h with 2θ varying between 15 and 130°, using copper radiation. The powder was polished for SEM observation and EDS measurements. The average composition measured from six points was Ca_2.1_Ce_7.9_(SiO_4_)_6_O_2_, which is close to the fixed composition of Ca_2_Ce_8_(SiO_4_)_6_O_2_ chosen for the refinement.

## Refinement   

**Figure 3:**
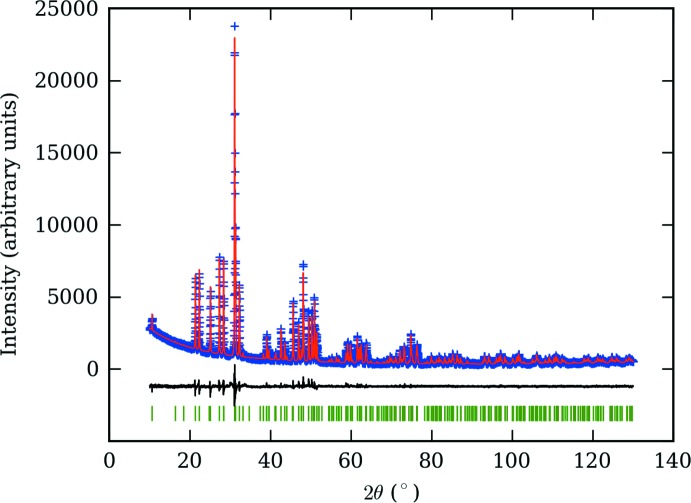
Experimental and calculated X-ray powder data profiles of Ca_2_Ce_8_(SiO_4_)_6_O_2_, with difference plot.

Details of the data collection and structure refinement are summarized in Table 3 ▸ and Fig. 3 ▸. The occupancies of the Si and O atoms were fixed to unity in agreement with the general observation that there are no vacancies on these sites for apatites. The total occupancies of the 6*h* and 4*f* sites were constrained to unity and the Ca_2_Ce_8_(SiO_4_)_6_O_2_ composition was kept fixed. The 6*h* site was considered as fully occupied by cerium since the refined calcium content was very low (0.9%). It is the same case as in the Pr-apatite structure (#00-029-0362) but not for the La (#00-029-0337) and Nd (#00-028-0228) apatites where calcium contents on the 6*h* site were determined to be 1.4% and 4%, respectively. The 4*f* site was modelled as half-occupied by Ca and Ce ions. Isotropic displacement parameters (*U*_iso_) were constrained to 0.008 Å^2^ for the 4*f* site atoms, 0.006 Å^2^ for the 6*h* site atoms, 0.005 Å^2^ for Si and 0.003 Å^2^ for oxygen sites. The residual electron density is 5.75 e Å^−3^ at a distance of 0.97Å from site Ce_*b*.

## Supplementary Material

Crystal structure: contains datablock(s) cacesio, I. DOI: 10.1107/S2056989018008435/wm5448sup1.cif

CCDC reference: 1848048

Additional supporting information:  crystallographic information; 3D view; checkCIF report

## Figures and Tables

**Table 1:** Selected bond lengths (Å) and angles (°) in the redetermined structure and the already published Ce apatite structure (Skakle *et al.*, 2000 ▸) and Ca_2_La_8_(SiO_4_)_6_O_2_

Bond length and angles	Skakle *et al.* (2000 ▸)	This work	La-apatite (Smith & McCarthy, 1977 ▸)
Ca/*Ln*—O1	2.51 (3)	2.468 (10)	2.485
Si1—O1	1.42 (5)	1.590 (16)	1.616
Si1—O2	1.59 (6)	1.614 (13)	1.625
Si1—O3	1.62 (3)	1.608 (7)	1.621
O1—Si1—O2	123 (2)	114.7 (7)	94.93
O1—Si1—O3	109.2 (21)	108.5 (5)	108.22
O2—Si1—O3	106.5 (20)	108.2 (5)	110.94
O3—Si1—O3	99.4 (17)	108.6 (4)	105.35
Distortion index of the SiO_4_ tetra­hedron*	0.04458	0.00396	0.00139

* as calculated by *VESTA* (Momma & Izumi, 2011 ▸).

**Table 2:** Cell parameters (Å) and volumes (Å^3^) for the title phase compared with isotypic Ca_2_ *Ln* ^III^_8_(SiO_4_)_6_O_2_ phases

*Ln*	Gd	Eu	Sm	Nd	Ce	Ce	La	Pr
Ref. PDF	28–0212	29–0320	29–0365	28–0228	This work	55–0835	29–0337	29–0362
*a*	9.421 (2)	9.440 (2)	9.466	9.529 (5)	9.59912 (6)	9.4343 (3)	9.651	9.565
*c*	6.888 (2)	6.918 (2)	6.949	7.022 (1)	7.09284 (6)	6.8885 (4)	7.151	7.060
*V*	529.4	533.9	539.2	552.2	566.00 (1)	530.98 (4)	576.8	559.4

**Table 3:** Experimental details

Crystal data	
Chemical formula	Ca_2_Ce_8_(SiO_4_)_6_O_2_
*M*_r_	1785.6
Crystal system, space group	Hexagonal, *P*6_3_/*m*
Temperature (K)	293
*a*, *c* (Å)	9.59912 (6), 7.09284 (6)
*V* (Å^3^)	566.00 (1)
*Z*	1
Radiation type	Cu *K*α_1_, λ = 1.540562, 1.544390 Å
Specimen shape, size (mm)	Flat sheet, 25 × 25

Data collection	
Diffractometer	Panalytical XPert MPD Pro
Specimen mounting	Packed powder pellet
Data collection mode	Reflection
Scan method	Step
2θ values (°)	2θ_min_ = 10.023 2θ_max_ = 130.010 2θ_step_ = 0.017

Refinement	
*R* factors and goodness of fit	*R*_p_ = 0.045, *R*_wp_ = 0.059, *R*_exp_ = 0.034, *R*(*F*) = 0.046, χ^2^ = 3.098
No. of parameters	28

Computer programs: *Data Collector* (Panalytical, 2011 ▸), *JANA2006* (Petříček *et al.*, 2014 ▸), *VESTA* (Momma & Izumi, 2011 ▸) and *publCIF* (Westrip, 2010 ▸).

## supplementary crystallographic information

### Crystal data

**Table:**

Ca_2_Ce_8_(SiO_4_)_6_O_2_	y
*M**_r_* = 1785.6	*D*_x_ = 5.239 Mg m^−^^3^
Hexagonal, *P*6_3_/*m*	Cu *K*α_1_ radiation, λ = 1.540562, 1.544390 Å
*a* = 9.59912 (6) Å	*T* = 293 K
*c* = 7.09284 (6) Å	Particle morphology: plate-like
*V* = 566.00 (1) Å^3^	brown
*Z* = 1	flat_sheet, 25 × 25 mm
*F*(000) = 796	Specimen preparation: Prepared at 1873 K and 100 kPa, cooled at 30 K min^−^^1^

### Data collection

**Table:**

Panalytical XPert MPD Pro diffractometer	Data collection mode: reflection
Radiation source: sealed X-ray tube	Scan method: step
Specimen mounting: packed powder pellet	2θ_min_ = 10.023°, 2θ_max_ = 130.010°, 2θ_step_ = 0.017°

### Refinement

**Table:**

*R*_p_ = 0.045	28 parameters
*R*_wp_ = 0.059	0 restraints
*R*_exp_ = 0.034	9 constraints
*R*(*F*) = 0.046	Weighting scheme based on measured s.u.'s
6881 data points	(Δ/σ)_max_ = 0.005
Profile function: pseudo-Voigt	Background function: Legendre polynoms

### Fractional atomic coordinates and isotropic or equivalent isotropic displacement parameters (Å^2^)

**Table:**

	*x*	*y*	*z*	*U*_iso_*/*U*_eq_	Occ. (<1)
Ca_a	0.333333	0.666667	−0.0009 (7)	0.008*	0.5
Ce_a	0.333333	0.666667	−0.0009 (7)	0.008*	0.5
Ce_b	0.23194 (13)	−0.01214 (18)	0.25	0.006*	
Si_c	0.4040 (6)	0.3713 (6)	0.25	0.005*	
O1_c	0.3293 (13)	0.4865 (12)	0.25	0.003*	
O2_c	0.5980 (14)	0.4634 (13)	0.25	0.003*	
O3_c	0.3410 (7)	0.2594 (8)	0.0668 (9)	0.003*	
O4_d	0	0	0.25	0.003*	

### Geometric parameters (Å, º)

**Table:**

Ca_a—Ca_a^i^	3.534 (7)	Ce_a—O1_c	2.468 (10)
Ca_a—Ca_a^ii^	3.559 (7)	Ce_a—O1_c^vi^	2.468 (8)
Ca_a—Ce_a	0	Ce_a—O1_c^vii^	2.468 (13)
Ca_a—Ce_a^i^	3.534 (7)	Ce_a—O2_c^iii^	2.437 (11)
Ca_a—Ce_a^ii^	3.559 (7)	Ce_a—O2_c^iv^	2.437 (10)
Ca_a—Si_c^iii^	3.246 (6)	Ce_a—O2_c^v^	2.437 (13)
Ca_a—Si_c^iv^	3.246 (8)	Ce_b—O3_c^viii^	2.396 (7)
Ca_a—Si_c^v^	3.246 (5)	Ce_b—O3_c^ix^	2.396 (7)
Ca_a—O2_c^iii^	2.437 (11)	Ce_b—O4_d	2.2870 (15)
Ca_a—O2_c^iv^	2.437 (10)	Si_c—O1_c	1.590 (16)
Ca_a—O2_c^v^	2.437 (13)	Si_c—O2_c	1.614 (13)
Ce_a—Ce_a^i^	3.534 (7)	Si_c—O3_c	1.600 (7)
Ce_a—Ce_a^ii^	3.559 (7)	Si_c—O3_c^ii^	1.600 (7)
			
Ca_a^i^—Ca_a—Ca_a^ii^	180.0 (5)	Ca_a^i^—Ce_a—O2_c^v^	43.5 (3)
Ca_a^i^—Ca_a—Ce_a	0	Ca_a^ii^—Ce_a—Ce_a^i^	180.0 (5)
Ca_a^i^—Ca_a—Ce_a^i^	0.0 (5)	Ca_a^ii^—Ce_a—Ce_a^ii^	0.0 (5)
Ca_a^i^—Ca_a—Ce_a^ii^	180.0 (5)	Ca_a^ii^—Ce_a—O1_c	43.9 (2)
Ca_a^i^—Ca_a—Si_c^iii^	57.01 (10)	Ca_a^ii^—Ce_a—O1_c^vi^	43.87 (19)
Ca_a^i^—Ca_a—Si_c^iv^	57.01 (11)	Ca_a^ii^—Ce_a—O1_c^vii^	43.9 (3)
Ca_a^i^—Ca_a—Si_c^v^	57.01 (9)	Ca_a^ii^—Ce_a—O2_c^iii^	136.5 (3)
Ca_a^i^—Ca_a—O2_c^iii^	43.5 (3)	Ca_a^ii^—Ce_a—O2_c^iv^	136.5 (3)
Ca_a^i^—Ca_a—O2_c^iv^	43.5 (3)	Ca_a^ii^—Ce_a—O2_c^v^	136.5 (3)
Ca_a^i^—Ca_a—O2_c^v^	43.5 (3)	Ce_a^i^—Ce_a—Ce_a^ii^	180.0 (5)
Ca_a^ii^—Ca_a—Ce_a	0	Ce_a^i^—Ce_a—O1_c	136.1 (2)
Ca_a^ii^—Ca_a—Ce_a^i^	180.0 (5)	Ce_a^i^—Ce_a—O1_c^vi^	136.13 (19)
Ca_a^ii^—Ca_a—Ce_a^ii^	0.0 (5)	Ce_a^i^—Ce_a—O1_c^vii^	136.1 (3)
Ca_a^ii^—Ca_a—Si_c^iii^	122.99 (10)	Ce_a^i^—Ce_a—O2_c^iii^	43.5 (3)
Ca_a^ii^—Ca_a—Si_c^iv^	122.99 (11)	Ce_a^i^—Ce_a—O2_c^iv^	43.5 (3)
Ca_a^ii^—Ca_a—Si_c^v^	122.99 (9)	Ce_a^i^—Ce_a—O2_c^v^	43.5 (3)
Ca_a^ii^—Ca_a—O2_c^iii^	136.5 (3)	Ce_a^ii^—Ce_a—O1_c	43.9 (2)
Ca_a^ii^—Ca_a—O2_c^iv^	136.5 (3)	Ce_a^ii^—Ce_a—O1_c^vi^	43.87 (19)
Ca_a^ii^—Ca_a—O2_c^v^	136.5 (3)	Ce_a^ii^—Ce_a—O1_c^vii^	43.9 (3)
Ce_a—Ca_a—Ce_a^i^	0	Ce_a^ii^—Ce_a—O2_c^iii^	136.5 (3)
Ce_a—Ca_a—Ce_a^ii^	0	Ce_a^ii^—Ce_a—O2_c^iv^	136.5 (3)
Ce_a—Ca_a—Si_c^iii^	0	Ce_a^ii^—Ce_a—O2_c^v^	136.5 (3)
Ce_a—Ca_a—Si_c^iv^	0	O1_c—Ce_a—O1_c^vi^	73.8 (4)
Ce_a—Ca_a—Si_c^v^	0	O1_c—Ce_a—O1_c^vii^	73.8 (4)
Ce_a—Ca_a—O2_c^iii^	0	O1_c—Ce_a—O2_c^iii^	94.4 (3)
Ce_a—Ca_a—O2_c^iv^	0	O1_c—Ce_a—O2_c^iv^	153.3 (4)
Ce_a—Ca_a—O2_c^v^	0	O1_c—Ce_a—O2_c^v^	126.7 (3)
Ce_a^i^—Ca_a—Ce_a^ii^	180.0 (5)	O1_c^vi^—Ce_a—O1_c^vii^	73.8 (4)
Ce_a^i^—Ca_a—Si_c^iii^	57.01 (10)	O1_c^vi^—Ce_a—O2_c^iii^	126.7 (4)
Ce_a^i^—Ca_a—Si_c^iv^	57.01 (11)	O1_c^vi^—Ce_a—O2_c^iv^	94.4 (3)
Ce_a^i^—Ca_a—Si_c^v^	57.01 (9)	O1_c^vi^—Ce_a—O2_c^v^	153.3 (6)
Ce_a^i^—Ca_a—O2_c^iii^	43.5 (3)	O1_c^vii^—Ce_a—O2_c^iii^	153.3 (3)
Ce_a^i^—Ca_a—O2_c^iv^	43.5 (3)	O1_c^vii^—Ce_a—O2_c^iv^	126.7 (5)
Ce_a^i^—Ca_a—O2_c^v^	43.5 (3)	O1_c^vii^—Ce_a—O2_c^v^	94.4 (4)
Ce_a^ii^—Ca_a—Si_c^iii^	122.99 (10)	O2_c^iii^—Ce_a—O2_c^iv^	73.2 (5)
Ce_a^ii^—Ca_a—Si_c^iv^	122.99 (11)	O2_c^iii^—Ce_a—O2_c^v^	73.2 (5)
Ce_a^ii^—Ca_a—Si_c^v^	122.99 (9)	O2_c^iv^—Ce_a—O2_c^v^	73.2 (3)
Ce_a^ii^—Ca_a—O2_c^iii^	136.5 (3)	O3_c^viii^—Ce_b—O3_c^ix^	139.4 (4)
Ce_a^ii^—Ca_a—O2_c^iv^	136.5 (3)	O3_c^viii^—Ce_b—O4_d	105.0 (2)
Ce_a^ii^—Ca_a—O2_c^v^	136.5 (3)	O3_c^ix^—Ce_b—O4_d	105.0 (2)
Si_c^iii^—Ca_a—Si_c^iv^	93.17 (18)	Ca_a^x^—Si_c—Ca_a^xi^	65.98 (17)
Si_c^iii^—Ca_a—Si_c^v^	93.17 (18)	Ca_a^x^—Si_c—O1_c	136.4 (2)
Si_c^iii^—Ca_a—O2_c^iii^	28.7 (3)	Ca_a^x^—Si_c—O2_c	46.6 (4)
Si_c^iii^—Ca_a—O2_c^iv^	64.6 (4)	Ca_a^x^—Si_c—O3_c	114.9 (5)
Si_c^iii^—Ca_a—O2_c^v^	97.0 (4)	Ca_a^x^—Si_c—O3_c^ii^	62.4 (3)
Si_c^iv^—Ca_a—Si_c^v^	93.17 (16)	Ca_a^xi^—Si_c—O1_c	136.4 (2)
Si_c^iv^—Ca_a—O2_c^iii^	97.0 (3)	Ca_a^xi^—Si_c—O2_c	46.6 (4)
Si_c^iv^—Ca_a—O2_c^iv^	28.7 (4)	Ca_a^xi^—Si_c—O3_c	62.4 (3)
Si_c^iv^—Ca_a—O2_c^v^	64.6 (3)	Ca_a^xi^—Si_c—O3_c^ii^	114.9 (5)
Si_c^v^—Ca_a—O2_c^iii^	64.6 (3)	O1_c—Si_c—O2_c	114.7 (7)
Si_c^v^—Ca_a—O2_c^iv^	97.0 (2)	O1_c—Si_c—O3_c	108.5 (5)
Si_c^v^—Ca_a—O2_c^v^	28.7 (3)	O1_c—Si_c—O3_c^ii^	108.5 (5)
O2_c^iii^—Ca_a—O2_c^iv^	73.2 (5)	O2_c—Si_c—O3_c	108.2 (5)
O2_c^iii^—Ca_a—O2_c^v^	73.2 (5)	O2_c—Si_c—O3_c^ii^	108.2 (5)
O2_c^iv^—Ca_a—O2_c^v^	73.2 (3)	O3_c—Si_c—O3_c^ii^	108.6 (4)
Ca_a—Ce_a—Ca_a^i^	0	Ce_a—O1_c—Ce_a^ii^	92.3 (5)
Ca_a—Ce_a—Ca_a^ii^	0	Ce_a—O1_c—Si_c	129.2 (3)
Ca_a—Ce_a—Ce_a^i^	0	Ce_a^ii^—O1_c—Si_c	129.2 (3)
Ca_a—Ce_a—Ce_a^ii^	0	Ca_a^x^—O2_c—Ca_a^xi^	93.0 (5)
Ca_a—Ce_a—O1_c	0	Ca_a^x^—O2_c—Ce_a^x^	0.0 (5)
Ca_a—Ce_a—O1_c^vi^	0	Ca_a^x^—O2_c—Ce_a^xi^	93.0 (5)
Ca_a—Ce_a—O1_c^vii^	0	Ca_a^x^—O2_c—Si_c	104.7 (4)
Ca_a—Ce_a—O2_c^iii^	0	Ca_a^xi^—O2_c—Ce_a^x^	93.0 (5)
Ca_a—Ce_a—O2_c^iv^	0	Ca_a^xi^—O2_c—Ce_a^xi^	0.0 (5)
Ca_a—Ce_a—O2_c^v^	0	Ca_a^xi^—O2_c—Si_c	104.7 (4)
Ca_a^i^—Ce_a—Ca_a^ii^	180.0 (5)	Ce_a^x^—O2_c—Ce_a^xi^	93.0 (5)
Ca_a^i^—Ce_a—Ce_a^i^	0.0 (5)	Ce_a^x^—O2_c—Si_c	104.7 (4)
Ca_a^i^—Ce_a—Ce_a^ii^	180.0 (5)	Ce_a^xi^—O2_c—Si_c	104.7 (4)
Ca_a^i^—Ce_a—O1_c	136.1 (2)	Ce_b^v^—O3_c—Si_c	146.2 (5)
Ca_a^i^—Ce_a—O1_c^vi^	136.13 (19)	Ce_b—O4_d—Ce_b^xii^	120.00 (5)
Ca_a^i^—Ce_a—O1_c^vii^	136.1 (3)	Ce_b—O4_d—Ce_b^xiii^	120.00 (6)
Ca_a^i^—Ce_a—O2_c^iii^	43.5 (3)	Ce_b^xii^—O4_d—Ce_b^xiii^	120.00 (6)
Ca_a^i^—Ce_a—O2_c^iv^	43.5 (3)		

Symmetry codes: (i) *x*, *y*, −*z*−1/2; (ii) *x*, *y*, −*z*+1/2; (iii) −*x*+1, −*y*+1, *z*−1/2; (iv) *y*, −*x*+*y*+1, *z*−1/2; (v) *x*−*y*, *x*, *z*−1/2; (vi) −*y*+1, *x*−*y*+1, *z*; (vii) −*x*+*y*, −*x*+1, *z*; (viii) *y*, −*x*+*y*, *z*+1/2; (ix) *y*, −*x*+*y*, −*z*; (x) −*x*+1, −*y*+1, *z*+1/2; (xi) −*x*+1, −*y*+1, −*z*; (xii) −*y*, *x*−*y*, *z*; (xiii) −*x*+*y*, −*x*, *z*.
